# Can DoloTest predict the efficacy of psychological treatment in patients suffering from severe headache?

**DOI:** 10.1186/1129-2377-14-S1-P146

**Published:** 2013-02-21

**Authors:** T Zimmer, D Kjeldgaard

**Affiliations:** 1 Danish Headache Center, Department of Neurology, Glostrup Hospital, University of Copenhagen, Denmark

## Objective

To test whether DoloTest [1], a new instrument for measurement of pain and quality of life, also is a valid test for documenting changes related to psychological treatment, in patients suffering from severe headaches.

## Method

DoloTest is a validated pain and quality of life assessment tool that has been applied to patients in different pain clinics, but newer tested in a headache clinic. It measures 8 different domains: Pain, problems with light physical activities, problems with more strenuous physical activities, problems doing your job, reduced energy and strength, low spirit, reduced social life and sleeping problems. The patient is asked to score an average of the past week on a VAS-scale for each domain. The maximum value of each domain score is 100 and 800 for the total score. The DoloTest provides a graphic picture of a patient’s pain, as well as a numeric score. Headache patients from the Danish Headache Center were referred to weekly psychoeducational group sessions within a cognitive framework including intensive relaxation training over a 9-weeks period, with 8 patients per group. Psychological treatment was given in addition to other multidisciplinary interventions. DoloTest was applied before, under and after the psychological treatment.

## Results

Baseline data for 110 patients, 22 males and 88 females, with an average age of 36.1 years (range 18 – 69) are collected. Most of the patients suffer from more than one type of headache: primarily migraine with or without aura n=66, frequent or chronic episodic tension-type headache n=70, others n=45. At baseline the average frequency of headache is 24 days/month, and the average total DoloTest score was 358 (range 32 – 675).

## Conclusion

The DoloTest may be at valuable tool to measure the effect of psychological treatment, and further evaluation is ongoing.
